# *Helicobacter pylori* overcomes natural immunity in repeated infections

**DOI:** 10.1002/ccr3.687

**Published:** 2016-09-23

**Authors:** Björn Stenström, Helen M. Windsor, Alma Fulurija, Mohammed Benghezal, M. Priyanthi Kumarasinghe, Kazufumi Kimura, Chin Yen Tay, Charlie H. Viiala, Hooi C. Ee, Wei Lu, Tobias D. Schoep, K. Mary Webberley, Barry J. Marshall

**Affiliations:** ^1^ Department of GastroenterologySir Charles Gairdner HospitalPerthWestern AustraliaAustralia; ^2^ The Marshall Centre for Infectious Diseases Research and TrainingSchool of Pathology and Laboratory MedicineThe University of Western AustraliaPerthWestern AustraliaAustralia; ^3^ Ondek Pty LtdQEII Medical CentrePerthWestern AustraliaAustralia; ^4^ Swiss Vitamin InstituteÉpalingesSwitzerland; ^5^ Department of Anatomical PathologyPathWest, QEII Medical CentrePerthWestern AustraliaAustralia; ^6^ Venasis Kanamachi Medical ClinicTokyo‐toJapan; ^7^ Telethon Kids InstitutePerthWestern AustraliaAustralia; ^8^ UM Marshall CentreUniversity of MalayaKuala LumpurMalaysia

**Keywords:** Helicobacter, natural immunity, reinfection, vaccination

## Abstract

Repeated experimental reinfection of two subjects indicates that *Helicobacter pylori* infection does not promote an immune response protective against future reinfection. Our results highlight the importance of preventing reinfection after eradication, through public health initiatives, and possibly treatment of family members. They indicate difficulties for vaccine development, especially therapeutic vaccines.

## Introduction

Billions of humans in the world are infected with *Helicobacter pylori* 1. While *H. pylori* infection typically leads to lifelong asymptomatic colonization of the stomach, 10% of infections result in peptic ulcers, 1–5% of infections result in gastric cancer, and more rarely, infection leads to mucosa‐associated lymphoid tissue (MALT) lymphoma 1, 2.

Treatment of *H. pylori*‐induced gastritis with antibiotics is effective 3. However, recurrence after treatment does occur 4. This could be due to failed eradication or reinfection. Our aim was to assess whether *H. pylori* infection in humans produces an immune response protective against future reinfection. Anti‐*H. pylori* antibodies are present in chronically infected persons, but fail to clear the infection 5. However, the protective role of natural immune responses to *H. pylori* has not previously been tested experimentally in humans.

To assess whether *H. pylori* could overcome natural immunity, we eradicated the asymptomatic *H. pylori* infection in otherwise healthy subjects and then rechallenged them with their own strain of *H. pylori*. We assumed immunity would be maximal against an individual's own strain and would lead to the failure of reinfection attempts.

Our findings have clinical significance in relation to strategies to combat *H. pylori*. They illuminate the significance of reinfection and inform vaccine development attempts, as an effective immune response is necessary for vaccination to work.

## Methods

Healthy volunteers (*n* = 312) were recruited through advertising. Three hundred and ten applicants were excluded (Table 1), leaving two largely asymptomatic adult subjects with *H. pylori* infection proven by C^14^ urea breath test (PYtest^®^ Kimberly Clark, USA) (UBT) 6.

**Table 1:** Reasons for exclusion of volunteers

Filtration reasons	No. of subjects
Excluded by interview (UBT not done)	
Pregnancy	0
Drug allergy (penicillin)	40
Regular use of contra‐indicated drugs: (aspirin, PPIs, NSAID, clopidogrel, warfarin)	45
Close contact with children aged 12 or younger at home or work	65
Enrolled in other studies	5
Have other gastric symptoms	20
Previously treated *Helicobacter pylori*	45
Pre‐existing medical condition	30
Aged over 75	4
History of stomach cancer in first‐degree relative	8
UBT negative for *H. pylori*	40
UBT positive for *H. pylori*	
Abnormal laboratory studies	2
Endoscopic abnormalities	2
Antibiotic‐resistant *H. pylori* strain	2
Declined further endoscopies	2
Total number of applicants excluded	310

**Figure 1:**
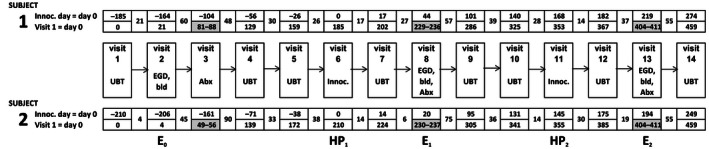
Timeline of the study. The study ran for approximately 15 months from baseline assessment and subsequent initial eradication and then through two reinfection and eradication cycles. Abx = antibiotic treatment (seven days), bld = blood test, UBT = urea breath test, Inoc. = inoculation (ingestion) of *H. pylori*, E_0_, E_1_, and E_2_ = endoscopy, HP_1_ and HP_2_ = ingestion of *H. pylori*.

Figure 1 provides a timeline. In brief, subjects underwent a baseline endoscopy with mucosal biopsies of the antrum and corpus taken for rapid urease test (CLOtest, Kimberly Clark, USA), *H. pylori* culture, histology, and immunohistochemistry. Culture and antibiotic susceptibility testing was performed as previously described 1. Isolates were screened for toxin genes (*cag*A and *vac*A) by PCR 5, 7. For histology, mucosal biopsies were fixed in 10% formalin and processed routinely. They were stained with hematoxylin and eosin (H&E) to assess morphology and toluidine blue to demonstrate bacteria.

Biopsies for immunohistochemistry were frozen at −80°C and later processed according to standard methods, with regulatory T cells enumerated 8. The specific antibodies used for immunohistochemistry were CD3‐ polyclonal, rabbit, Dako; CD4+ ‐ SP35, monoclonal, rabbit, Cell Marque; FoxP3‐SP97, Spring Bioscience, rabbit. FoxP3+ and CD4+ T cells from mucosal biopsies were counted and compared to overall T‐cell (CD3+) populations. T cells were enumerated manually across five independent microscopic fields at 40x magnification, and averages reported.

Blood samples were also collected at this and subsequent endoscopies and used to monitor liver function, kidney function, electrolytes, and full blood count. Processing of samples was performed in accordance with the standard practice at PathWest (a certified laboratory).

Serum samples were evaluated by ELISA (IgG) as previously described 9.

At the initial (and final endoscopy), endoscopic gastric secretion tests (EGT) 10 were conducted: Gastric acid secretion over a 10‐min period was measured after subcutaneous pentagastrin injection, followed by a 20‐min delay. The EGT value was multiplied by six to provide a value equivalent to maximal acid output (MAO).

After the initial endoscopy, there was a delay to ensure successful bacterial culture. Subjects then took a seven‐day eradication therapy consisting of esomeprazole (20 mg), amoxicillin (1000 mg), and clarithromycin (500 mg) twice daily. Tinidazole (500 mg) twice daily was added for days five to seven. Eradication was confirmed by two UBTs at least 4 weeks apart. Subsequently, the subjects drank a dose of 10^9^ colony‐forming units of *H. pylori* culture suspended in 10 mL of commercially available beef broth. This followed ingestion of 40 mg of famotidine the night before. Two weeks later, infection status was determined by UBT. Subjects then underwent another endoscopy with biopsy collection followed by eradication therapy. At least 124 days post‐therapy, subjects again drank their *H. pylori* and repeated the cycle.

Diary cards were used to record symptoms for 14 days at baseline, for 7 days during each antibiotic treatment, and for 14 days after each reinfection.

Written informed consent was given by the subjects. The study was approved by the Sir Charles Gairdner Hospital Human Research Ethics Committee (study number 2007‐045). The trial is registered with the Australia New Zealand Clinical Trials Registry 11 (trial number ACTRN12607000467437).

## Results

### Baseline results

Baseline investigation confirmed that subjects had *H. pylori* infections, but did not have other significant pathology or antibiotic‐resistant *H. pylori*.

Subject 1, a 58‐year‐old female, experienced occasional heartburn prior to the study. Subject 2, a 30‐year‐old male, was completely asymptomatic. Initial endoscopic examination of subject 1 showed a small hiatus hernia, two tiny erosions at the gastroesophageal junction, erythema of the gastric antral mucosa, and petechiae in the duodenum. Erythema plus mild nodularity of the proximal antrum was reported in subject 2.

**Figure 2:**
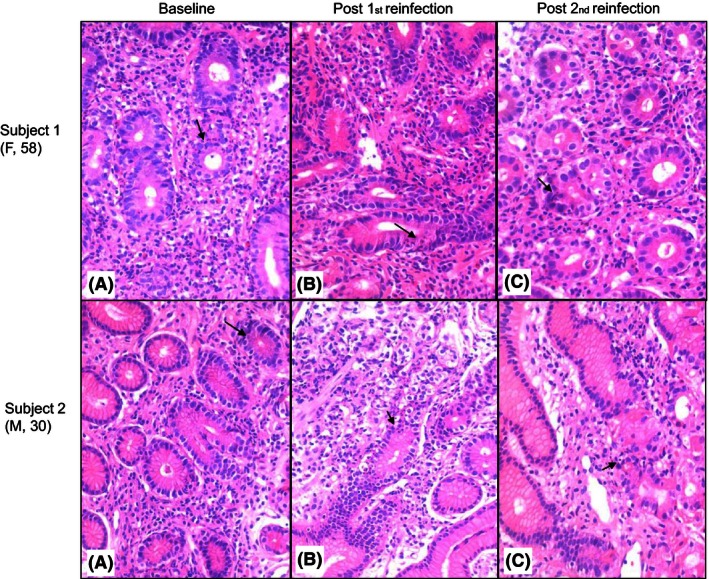
Histology from endoscopic biopsies taken from subjects 1 and 2 at baseline (A), post the first reinfection (B), and post the second reinfection (C), all featuring *H. pylori*‐associated active chronic gastritis (hematoxylin & eosin x400 magnification). The arrows indicate activity.

Gastric mucosal biopsies of both subjects showed active chronic gastritis with *H. pylori* (Fig. 2, Table 2), and all samples were also positive for *H. pylori* by histology, RUT, and culture (Table 2).

**Table 2:** Results from endoscopic examinations and diagnostic tests

Visit	1	2	3	4	5	6	7	8	9	10	11	12	13	14
Action	Baseline	EGD	Eradication			Reinfection		EGD then eradication			Reinfection		EGD then eradication	
Subject 1														
Day	0	21	81	129	159	185	202	229	286	325	353	367	404	459
UBT (DPM)	**2189**			0	29		**2298**		2	6		**2328**		0
Endoscopy (esophagus)		Erosions						Normal					Normal	
Endoscopy (stomach)		Erythema						Erythema + erosions					Erythema	
Endoscopy (duodenum)		Erythema						Normal					Normal	
Histology		*Hp‐*pos						*Hp‐*pos					*Hp‐*pos	
RUT		Pos						Pos					Pos	
Culture		Pos						Pos					Pos	
Subject 2														
Day	0	4	49	139	172	210	224	230	305	341	355	385	404	459
UBT (DPM)	**1987**			0	0		**1108**		3	7		**1977**		6
Endoscopy (esophagus)		Normal						Normal					Normal	
Endoscopy (stomach)		Erythema + nodularity						Erythema					Erythema + nodularity	
Endoscopy (duodenum)		Normal						Normal					Normal	
Histology		*Hp‐*pos						*Hp‐*pos					*Hp‐*pos	
RUT		Pos						Pos					Pos	
Culture		Pos						Pos					Pos	

The study ran for approximately 15 months. Examinations and tests were carried out at key points from baseline assessment, after initial eradication, and then through two reinfection and eradication cycles, for each subject. The diagnostic tests were urea breath test, histological study of gastric biopsies stained with toluidine blue to demonstrate bacteria, rapid urease test on biopsies, and culture from biopsies. The UBT results are presented in bold where positive for *Helicobacter pylori* infection (>199dpm).

EGD, Esophagogastroduodenoscopy; UBT, diagnostic urea breath test; RUT, rapid urease test, eradication = 7 days of antibiotics.

PCR studies showed that subject 1's strain was *cag*A+ with the *vac*A s1 m2 subtype, while subject 2's strain was *cag*A‐ and had the *vac*A s2 m2 subtype.

### Eradication and reinfection cycles

Subjects were proven free of *H. pylori* after the first antibiotic treatment by UBT (Table 2). Subjects were then re‐infected with their own *H. pylori* strain. Reinfection was confirmed by UBTs after two weeks and at endoscopy (Table 2, Fig. 2). A second antibiotic treatment was given, and subjects were proven free of *H. pylori* by UBT (Table 2).

Subjects underwent a second cycle of reinfection proven by UBT and biopsies (Table 2, Fig. 2). Final eradication of *H. pylori* was confirmed by UBT.

### Clinical symptoms

Subject 1 reported increased heartburn after the second reinfection. She noted softer stools after both reinfections, and diarrhea, for 2 days, and vomited once after the second reinfection. Subject 2 reported halitosis after both reinfections.

### Blood chemistries

Blood chemistry remained within the normal range for both subjects.

### Immunological studies

**Figure 3:**
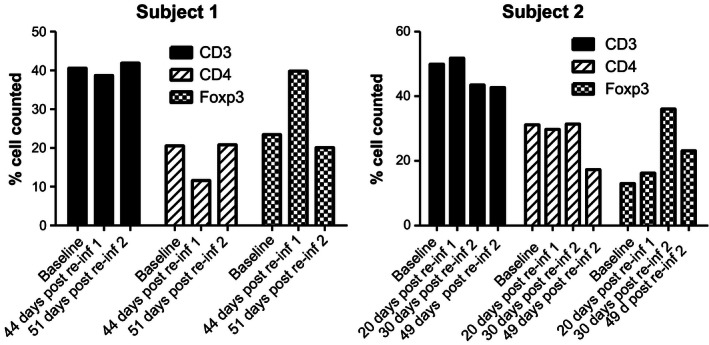
Stomach biopsies were collected from each subject at the indicated visits. Numbers of FoxP3+, CD4+, and CD3+ T cells were determined by IHC using specific antibodies. Cells were enumerated by visual counts, and results shown are the mean of the % count of five separate fields for each cell type.

**Figure 4:**
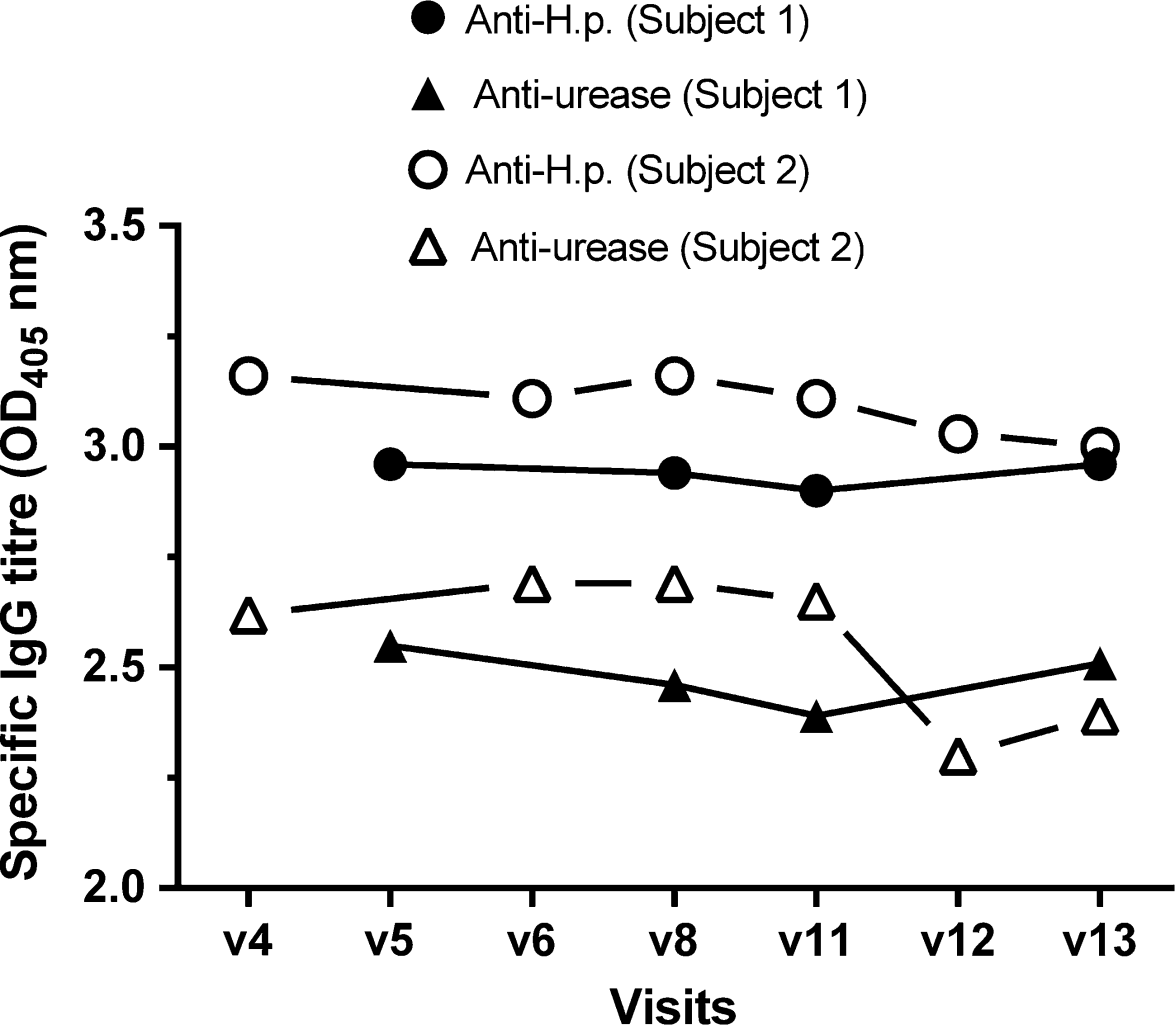
Anti‐Helicobacter IgG response in two human subjects re‐infected with *H. pylori*. Serum samples were collected from each subject at the indicated visits. Anti‐*H. pylori* and anti‐*H. pylori* urease IgG antibodies were measured by standard ELISA. Anti‐*H. pylori* IgG and anti‐*H. pylori* urease IgG titers at different time points in the study for subject 1 and subject 2. Results are expressed as the optical density (OD) at 405 nm from sera diluted at 1:20. The anti‐*H. pylori* IgG response was stable across the study and did not decline after the eradication of *H. pylori*. The anti‐urease IgG response reflects this observation. *H. pylori* antibodies persist during chronic infection.

Regulatory T cells (13–25%) were present in both subjects at baseline (Fig. 3) and typically increased upon reinfection. Total T‐cell numbers remained stable. Subjects showed persistent antibody titers throughout the study (Fig. 4).

### Gastric acid secretion

Gastric acid secretion was within normal ranges (normal range for MAO is 5–30 mmol/h in women and 7–47 mmol/h in men 12. At initial endoscopy, MAO was calculated as 37.1 mEq/h for subject 1 and 27.4 mEq/h for subject 2. At visit 13, it was 28.1 mEq/h for subject 1 and 33.9 mEq/h for subject 2.

## Discussion

We showed that, in two subjects with prior *H. pylori* infection and high levels of *H. pylori*‐specific antibodies, reinfection was possible on two occasions. These cases demonstrate, for the first time experimentally, that natural immunity in humans is not protective.

The results are consistent with epidemiological studies 4. Recurrence of infection has been observed at low‐to‐moderate levels in various populations. However, differentiation between recrudescence due to treatment failure and reinfection has been problematic. Even where molecular fingerprinting techniques confirm the bacteria are the same over time, one cannot differentiate between failed therapy and reinfection from a family member with the same strain 6. Study of the rate of recurrence over multiple years suggests that while recrudescence may explain the majority of cases in the developed nations, and reinfection is more common in developing populations 6. Our study supports the view, as it provides direct evidence to prove that reinfection is possible. The results are also congruent with the successful experimental reinfection in monkeys 13, 14. However, our study is unique, not only because it was in humans, but also because we tested immunity to the original strain. Deliberate infection in humans had previously only been performed with a strain from an external source (BCS100) 15, 16.

During our study, a sustained immune regulatory environment was maintained with high levels of regulatory T cells. This offers a potential mechanism for the lack of protection. Similarly, regulatory T cells are elevated during chronic infection and aid the bacteria to survive in the host 8, 17. However, determining the exact mechanism requires further study, as the bacterium is known to exhibit a complex array of evasion strategies 18, 19.

When might natural immunity be effective? In natural infection, exposure to numerous *H. pylori* strains probably occurs in childhood until the subject is colonized by a strain suited to that individual's gastric mucosa. It may be able to evade the host's immune system because of a particular combination of bacteria, host, or environmental factors. If so, it could be that our subjects always remained susceptible to their own strains, but would be able to eliminate others.

Other reports indicate that innate immune responses may offer protection and that acute infections are more likely to be cleared than chronic infections. Marshall spontaneously cleared his *H. pylori* infection, despite failing to seroconvert 20. Similarly, Okuda et al. found a high (75%) rate of transient infections in infants (but could not rule out false positives) 21.

Our findings have implications for public health strategies to control *H. pylori*. As eradication therapy does not prevent future reinfection, antibiotic use needs to be combined with measures to stop reinfection. New epidemiological research is needed to determine the key transmission routes. There is evidence for and against a range of routes (see Mitchell 2001 22 for a review). Development of screening techniques that allow isolation and culture of *H. pylori* from contaminated specimens from the environment and subjects is vital to further this research 22. In the meantime, we suggest that eradication should be combined with education to improve hygiene, provision of clean water supplies and sanitation, and possibly synchronized eradication across families. This accords with Statement 7 of the Second Asia‐Pacific Guidelines for *H. pylori* infection, which states that in areas of high prevalence, eliminating *H. pylori* infection through improvements in public health and education will have the greatest impact in reducing the burden of gastric cancer 23. The statement focuses on the benefits of preventing first infections in patients who may never be treated, but our results also highlight the problem of reinfection.

The obvious alternative to antibiotics is vaccination. Unfortunately, the ability of *H. pylori* strains to repeatedly infect humans despite the presence of antibodies is reason for pessimism concerning the development of a therapeutic *H. pylori* vaccine. Vaccination against a high burden of *H. pylori*, as is the case in therapeutic vaccination, will be difficult to achieve, probably because of the down‐regulated immune environment (35, 36). Our results are in accordance with reviews lamenting failure to develop an effective *H. pylori* vaccine 2, 24, 25. However, recently, Zeng et al. reported the success of a prophylactic *H. pylori* vaccine in a field trial in school‐aged children 26. This demonstrates that under conditions of low bacterial burden and acute infection, *H. pylori* infection may be prevented. Certainly, the trial of Zeng et al. represents an extremely promising advance. However, as Sutton has outlined there is still some way to go before reaching a marketable vaccine 27. We agree with the Maastrict IV/Florence Consensus Report (Statement 20) that the profound benefits of an *H. pylori* vaccine merit continued effort in this area 28. Our results and others 29 indicate that protection independent of B‐cell responses should be considered, with a focus on prophylactic vaccination.

While host immune regulation by *H. pylori* may be challenge for vaccine development, it may have potential benefits 29. The immune modulatory properties of *H. pylori* may be exploited in the fight against inflammatory diseases such as allergies. Similarly, exploiting *H. pylori's* ability to evade the immune system may permit the use of safe genetically modified strains as a live delivery agent 30 that can be used repeatedly.

The study had several limitations. It was limited to two subjects as asymptomatic adults with *H. pylori* were hard to find. The scarcity of *H. pylori* ‐infected volunteers in Western Australia has previously been reported 31.

A larger study would increase generalizability of the results and could include subjects with greater symptoms indicative of immune response. These patients may not be so susceptible to reinfection. In addition, infection with novel strains would test whether subjects are able to eliminate other strains, even while susceptible to their own. However, this question may be better addressed in animal models, given the greater risk of harm. Safety was a priority in our study. Hence, we started with largely asymptomatically infected subjects and only reinfected them with their own strains. If we could not eradicate *H. pylori* after reinfection, they would not be in a worse state than before the study.

We hypothesize that the presence of a strong immune regulatory environment contributed to successful reinfection. However, our immunological studies were only preliminary, and more comprehensive assays at more time points are necessary.

As we assumed reinfection might be difficult, we used high doses of bacteria. The minimum bacterial dose required to reinfect a person was not determined.

## Conclusion

We have shown in two subjects that the natural immune response to *H. pylori* does not protect against reinfection with the same strain. Our findings highlight the importance of avoiding reinfection from the environment and from family members following eradication. The immune regulatory properties of *H. pylori* and the lack of natural immunity suggest that therapeutic vaccination against *H. pylori* will be difficult. However, prophylactic vaccines may be possible.

## Conflict of Interest

The Authors disclose the following: Grants from Ondek Pty Ltd. BJM is a shareholder in Ondek Pty Ltd, and MB, AF, WL and TDS were employed by from Ondek Pty Ltd at the time of the study and at other times. In addition, several of the authors (BJM, AF, WL, AF, TDS & MB) have patents pending or issued to Ondek Pty Ltd including patents relating to *Helicobacter pylori* and immunotherapy.
